# The health of lesbian, gay, bisexual, transgender and intersex people

**DOI:** 10.25646/6449

**Published:** 2020-03-18

**Authors:** Kathleen Pöge, Gabriele Dennert, Uwe Koppe, Annette Güldenring, Ev B. Matthigack, Alexander Rommel

**Affiliations:** 1 Robert Koch Institute, Berlin Department of Epidemiology and Health Monitoring; 2 Fachhochschule Dortmund – University of Applied Scienes and Arts, Social medicine and public health with a focus on gender and diversity; 3 Robert Koch Institute, Berlin Department of Infectious Disease Epidemiology; 4 Westküstenkliniken Heide/Brunsbüttel, Department of Psychiatry, Psychotherapy and Psychosomatics; 5 German chapter of the International Association of Intersex People (IVIM), Organisation Intersex International (OII Germany), Berlin

**Keywords:** HEALTH, SEX AND GENDER, INTERSEX, TRANSGENDER, SEXUAL ORIENTATION

## Abstract

Sex, gender and sexual orientation are diverse, as are the ways of living associated with them. The extent to which people can live a free and self-determined life according to their own body, gender, sexuality and way of life influences their social resources, opportunities for participation and discrimination and has an influence on their life situation and health. A narrative review of lesbian, gay, bisexual, transgender and intersex (LGBTI) health was conducted including international and German reviews, meta-analyses and population-based studies. The focus of this article is the legal, social and medical recognition as well as health status of LGBTI people in Germany. While the legal framework in Germany for homosexual and bisexual people has gradually improved, many civil society stakeholders have pointed to major deficits in the medical and legal recognition of transgender and intersex people. In addition, scientific findings frequently have not yet found its way into medical practice to an adequate extent. Available data on LGBTI health indicate a need for action in the areas of mental health and health care provision. However, due to a lack of comprehensive data, conclusions cannot be drawn on the general health situation and health resources of LGBTI people. For the concrete planning and implementation of measures as well as the differentiated portrayal of the situation in Germany, the databases must be expanded, not least via population-representative surveys.

## 1. Introduction

The diversity of sexes, genders, sexual orientations and ways of living is part of the diversity of society. Although developments must be viewed as differentiated and are in many areas only in their infancy, lesbian, gay, bisexual, transgender and intersex people (LGBTI, Info box Annex) have gained some social and legal recognition over the past years. This can be seen, for example, in the 2017 law allowing marriage for same-sex couples, and in the German Civil Status Act from 2018 allowing intersex people to list their sex/gender marker as ‘diverse’ instead of ‘female’ or ‘male’, or leaving the field blank.

The degree to which people can live a free and self-determined life according to their own body, gender, sexuality and way of life influences their social resources, opportunities for participation and discrimination and has an influence on their life situation and health. It is not an individual’s sex, gender identity or sexual orientation that can lead to different health outcomes from the overall population, but rather the determining factor of social context. The predominant social model of the heterosexual sex/gender binary can have a negative impact on the lives and health of lesbian, gay, bisexual, transgender and intersex people. Conversely, an increase in recognition of diversity may translate into greater life satisfaction and health.

Although there are many similarities between the health needs of LGBTI people and the overall population, there are also LGBTI-specific issues. The self-reported physical health of lesbian, gay and bisexual people, for example, does not differ from that of heterosexual people [1]. Regardless of sexual orientation, the great majority of people enjoy either good or very good subjective health. However, international studies have also shown that depression and suicidal behaviour are more common among lesbian, gay, bisexual, transgender and intersex people [1–5]. In this context, the individual and social resources available to a person are significant. For example, it has been shown that the mental health of homosexual, bisexual and transgender people is better if they can express their sexual orientation and/or gender identity without discrimination and can rely on a supportive social and family environment [6–8].

The present article provides an overview of the social situation and health status of LGBTI people and indicates where action is most urgently needed. Initially, we discuss the number of LGBTI people living in Germany and identify the social, legal and medical frameworks in which they find themselves. We then describe the health situation of LGBTI people and the healthcare they receive based on findings from German and international scientific findings. Finally, efforts to improve the information base in the field of epidemiology and survey research are discussed that aim at further developing the evidence for health and social reporting.

When interpreting the results, it must be noted that existing research often focuses on particular topics such as the sexual health of gay men and the mental health of LGBTI people in general. Little consideration has been given until now to the general health status of LGBTI people and the factors that benefit their health. It should therefore be noted that this review is based on a selective presentation of certain topics. Data gaps and limitations are identified in the discussion, which also presents an outlook on positive developments regarding the information base on LGBTI health.

## 2. Methodology

This article is based on a literature research on the health of LGBTI people from the PubMed and Web of Science databases as well as other relevant web sites. Studies on the situation in Germany were included, as well as international English-language reviews, meta-analyses, and population and community-based studies published between 2000 and January 2020. The size of Germanys LGBTI population was estimated based on published findings from population-based surveys, namely the German Socio-Economic Panel (SOEP) from 2016 [9], the Microcensus from 2015 [10] and the Federal Centre for Health Education (BZgA) study on Youth Sexuality in Germany from 2015 [11]. The description of the legal framework was based on resolutions and legal documents from Germany and abroad.

## 3. Proportion of LGBTI people in the population

The proportion of the population of lesbian, gay, bisexual, transgender and intersex people living in Germany can only be roughly estimated.

### 3.1 Homosexuality and bisexuality

Sexual orientation can be distinguished into three dimensions: sexual identity (e.g. heterosexual, homosexual or bisexual), sexual desire or attraction, and sexual behaviour. These three dimensions are not necessarily in line with one another and can change throughout a person’s life [12]. In 2016, the SOEP gathered voluntary self-reported data on sexual orientation. On the basis of this data and other sources, it was estimated that around 2% of the German population identifies as lesbian, gay or bisexual (Table 1). This number is lower in the 60+ age group than for those under the age of 45. BZgA findings also indicate that younger people are more likely to identify as lesbian, gay or bisexual (Table 1). There are also differences between men and women, in that women identify slightly more frequently as bisexual, and slightly less frequently as lesbian, whereas men describe themselves slightly more frequently as gay and slightly less frequently as bisexual [13]. Overall, only a small group clearly identifies as lesbian, gay or bisexual. A significantly higher proportion of people describe themselves as not exclusively heterosexual reaching double-digit values for women and men in various studies (Table 1). The figures also show that a higher proportion of people feel attracted to people of the same sex (desire/attraction) or have had same-sex sexual contact (behaviour) than people who identify explicitly as homosexual or bisexual (identity) (Table 1). The figures increase with age and as well reach two-digit values in young adulthood (Table 1) [11].

According to Microcensus data, the number of samesex couples who live together increased by 57% from 60.000 to 94.000 between 2005 and 2015 (Table 1). However, not all homosexual and bisexual people live in samesex partnerships and it must be taken into account that self-reporting underestimates reality due to fears of stigmatisation [14].

In addition, SOEP data allows to identify social differences between lesbian, gay and bisexual people on the one hand and heterosexual people on the other. More than half of those who identify as lesbian, gay or bisexual live in cities with more than 100.000 inhabitants, compared to one-third of heterosexuals [1]. Generally, they are less likely to live in a partnership and more likely to rely on support networks outside their family than heterosexual people. Moreover, lesbian, gay and bisexual people on average tend to reach a higher level of education. However, according to SOEP data, gay men in particular earn significantly less than heterosexual men [1, 16]. Over the course of a lifetime, this can lead to fewer financial resources and poverty in old age [17].

### 3.2 Transgender and intersex

Estimating the size of Germany’s intersex and transgender population is considerably more difficult. On the basis of the country’s Transsexual Act (Transsexuellen-Gesetz, TSG), some transgender people apply to have their sex/gender entry and first name changed. Between 2008 and 2016, the number of these applications increased from 903 to 1,868 per year [18]. An international meta-analysis has calculated there are 4.6 transgender people per 100,000 people [19]. In 2015, the German Medical Association released a statement that around 150 children are born with a ‘disorder of sex development (DSD)’ each year [20]. According to the Ethics Council of Germany, around 80,000 intersex people are estimated to live in Germany [21]. A review of scientific and clinical studies suggests that between 0.018% and 2.1%, or 3.8% of all births exhibit ‘variants of sex development’ or the urogenital system [22]. The United Nation’s Free & Equal initiative assumes that between 0.05% and 1.7% of the population are intersex [23, 24]. However, these figures should be considered a rough approximation as there are no reliable data on the number of transgender and intersex people in Germany.

## 4. Social and legal framework

Social movements and LGBTI civil society organisations have encouraged a positive self-image and positive external perception of LGBTI people. They have also made a key contribution to increasing social recognition. However, despite various advances, there are still deficits in social and legal equality and in medical recognition.

### 4.1 The heteronormative orientation of society

**Figure 1:**
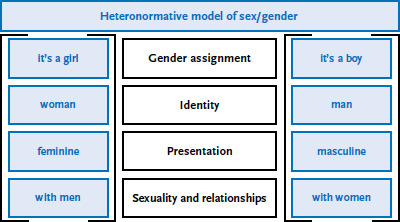
Heteronormative model of sex/gender Source: Rommel et al. 2019 [26]

The heteronormative orientation of society can pose a health risk for LGBTI people. The concept of heteronormativity conveys the idea that there are only two sexes which are each congruent with two genders (women and men) which are sexually oriented towards one another [25]. Heteronormativity is expressed in normative social expectations that intersex, transgender and non-heterosexual people do not fulfil (Figure 1).

Heteronormativity can express itself socially in the non-recognition of congenital variations of sex characteristics, sexual identity, self-representation of gender or sexual orientation. This lack of recognition can range from prejudice and discrimination to physical and sexualised assault [27]. It is also inscribed in legal regulations that deny LGBTI people certain rights. A number of studies from the US, the UK and Australia show that homosexual, bisexual and transgender adolescents frequently experience harassment [28, 29]. A study by the German Youth Institute (DJI) on coming out experiences (Info box Annex) found that 73.9% of the lesbian, gay, bisexual and transgender adolescents and young adults surveyed feared being rejected by their friends, 69.4% by their family and 20.2% reported fearing physical violence [30]. Transgender and intersex people report discrimination linked to the expression of their gender in their everyday lives, in education and in the labour market [31, 32].

These experiences as part of a gender or sexual minority can cause stress and consequently have a negative effect on physical and mental health as well as health behaviour (Minority Stress Model [33], Psychological Mediation Framework [34]). The pejorative use of the terms ‘lesbian’ and ‘gay’, for example, can lead to negative, internalised attitudes toward homosexuality (internalised homonegativity [35]). These attitudes can cause low self-acceptance and self-degradation in lesbian, gay and bisexual people, encouraging feelings of loneliness and negatively affecting mental health [30]. The LGBTI communities and civil society organisations, which crucially enable contact as well as offering networking opportunities and advice, are an important resource for dealing with discriminatory experiences and for developing a positive self-image. Contact with people with similar life experiences and interests, as well as shared activities, can have a positive effect on psychological well-being [36].

### 4.2 Legal recognition and equality

The way in which society deals with sex, gender and sexual diversity is also a question of human rights. The United Nations (UN) and Council of Europe human rights convention ratified by Germany ensures that all people have an equal opportunity to participate in society. On an international level, UN resolutions have been demanding recognition of gender and sexual minority rights and an end to criminal prosecution for years [37]. Article 3 of the German Constitution (Grundgesetz) stipulates equal treatment by the state and the 2006 General Act on Equal Treatment (Allgemeines Gleichbehandlungsgesetz) prohibits any form of unequal treatment in connection to a person’s gender and sexual identity in labour and civil law.

For many years, homosexual women and men in Germany were systematically denied certain rights and were subject to persecution. Gay men were criminalised when West Germany adopted paragraph 175 of the penal code (‘fornication between men’), a provision which had been strengthened by the National Socialists. In the German Democratic Republic (GDR), the paragraph was effectively invalidated in 1957 and finally abolished in 1968. However, these legal developments in the GDR should not be equated with a generalised social acceptance of homosexuality. Paragraph 175 was abolished in West Germany in 1994 after the German reunification.

For a long time, non-heterosexual partnerships were not legally recognised and partners could not assert mutual rights and obligations. This changed with the introduction of registered civil partnerships in 2001 and the extension of marriage to include same-sex couples in 2017. This also enabled the right of same-sex partners to jointly adopt non-biological children.

In 2011, the Ethics Council of Germany held a hearing on the situation in Germany for intersex people [38]. Changes to the Civil Status Act in 2013 made it possible for the first time to leave the sex/gender entry blank. Since the end of 2018, it is no longer mandatory to record the sex/gender of intersex children, parents may choose to or not as they wish. The new Civil Status Act, which gives intersex people a choice of ‘diverse’ as well as ‘female’, ‘male’, or leaving the field blank, created a new legal situation. The underlying constitutional court ruling from 2017 recognized for the first time the existence of sexes/genders beyond ‘female’ and ‘male’ [39]. However, intersex people are still required to provide a medical certificate before being allowed to change their sex/gender entry, a procedure which many intersex people find discriminatory.

Although the amendment to the law was designed for intersex people, transgender people also try to use this regulation to change their sex/gender entry. Transgender people regard the Transsexual Act from 1980 as discriminatory in many respects. A change of first name and sex/gender entry must be confirmed by two expert opinions paid for by the applicant as well as a court decision [40]. Until 2011, the Transsexual Act also required transgender people to undergo surgery resulting in an ‘inability to reproduce’ if they wanted to have their sex/gender entry changed. A ruling by the Federal Constitutional Court in 2011 removed this major barrier to reproductive health for transgender people, yet many are calling for the removal of other barriers that make changing first names and registered sex/gender difficult [41].

**Figure 2:**
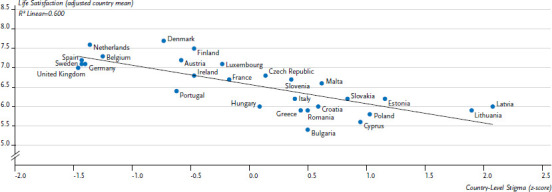
Self-reported life satisfaction among sexual minorities in Europe by degree of structural stigma at country level Source: Pachankis & Branstrom (2018) [42]

The degree to which sex, gender and sexual diversity are legally recognised has a significant effect on equal opportunities for social participation and health [42, 43]. It determines what resources and opportunities are available for social participation of lesbian, gay, bisexual, transgender and intersex people, as well as what discrimination they face. A comparative international study has demonstrated the connection between changing social frameworks on the one hand and wellbeing and health on the other [42]. Societal frameworks are measured using the concept of structural stigma. To measure this concept, measure was created based on relevant laws and political measures collected continuously by the European branch of the International Lesbian, Gay, Bisexual, Trans and Intersex Association (ILGA) and on attitudes towards lesbian and gay people in the population taken from the European Social Survey [42]. The findings not only show that the degree of structural stigma differs significantly between European Union countries, but also that a greater degree of structural stigma is linked both to a tendency to disguise homosexual and bisexual orientations [42] and to a lower level of life satisfaction for those affected (Figure 2). A similar pattern can be seen among US federal states. The mental health of lesbian, gay and bisexual people is better in states that allow same-sex marriage [44].

The degree of structural stigma in Germany is relatively low compared to other countries. However, despite legal advances, the reality for sex/gender and sexual minorities is still marked by disadvantage. To further promote the equality of LGBTI people under the rule of law provides an essential foundation for a self-determined life and strengthens social recognition.

### 4.3 Medical recognition

The past years have seen an increase in the medical recognition of sex, gender and sexual diversity, with decreasing levels of pathologisation by healthcare professions and their associations. However, there is still a need for action, particularly in regard to transgender and intersex people. An important step for the recognition of sexual diversity was made in 1990 when the General Assembly of the World Health Organization (WHO) removed homosexuality from the list of mental illnesses [45]. At the end of 2019, the German federal cabinet banned interventions aimed at changing or suppressing a person’s sexual orientation or self-perceived gender identity (so-called conversion therapies) on the grounds that this type of intervention often causes psychological damage. From 2020, the ban will apply to minors and adults incapable of giving consent and will also affect the public advertising, provision and mediation of such measures with the aim of safeguarding gender and sexual self-determination.

Until now, the WHO’s International Statistical Classification of Diseases and Related Health Problems, 10th revision (ICD-10), classifies ‘transsexualism’ (F64.0) as a mental disorder. The 11th revision of this classification (ICD-11), which was adopted by the WHO in 2019 and will come into effect in 2022, no longer defines being transgender as a mental disorder, but instead as ‘gender incongruence’ in the category ‘sexual health conditions’. ‘Gender incongruence’ describes identification with either a gender other than that assigned at birth or with no gender [46], and thus a discrepancy between a person’s perceived gender and their sex characteristics. This is no longer classified as a psychological illness per se [47], although according to the fifth edition of the Diagnostic and Statistical Manual of Mental Disorders (DSM-5) it can, under certain circumstances, lead to a mental disorder that has been termed ‘gender dysphoria’ since 2013 [48].

In 1987, the Federal Social Court of Germany ordered health insurance providers to cover the costs of sex reassignment surgery for transgender people. In 1997, the German Society for Sexual Research (DGfS), the Academy of Sexual Medicine (ASM) and the Society of Sexology (GSW) published the German ‘Standards for the Treatment and Diagnostic Assessment of Transsexuals’ (Standards der Behandlung und Begutachtung von Transsexuellen). These standards define the psychiatric and psychotherapeutic treatment processes, the process of diagnosis and the timeframe for treatment indications of sex reassignment measures. Based on these standards, the Medical Commission of the National Association of Statutory Health Insurance Funds (MDS) published a binding ‘Guideline for the Assessment of Transsexuality’ (Begutachtungsanleitung Transsexualität) in 2009 [49], which is standardised according to a binary gender concept and still remains in force. This guideline limits the ability of doctors to provide individual and autonomous treatment options [49]. The MDS requires a so-called Real-Life Test [50] designed to evaluate the ‘liveability’ of the new sex/gender, in addition to 18 months of psychotherapy.

The ‘Guideline for the Assessment of Transsexuality’ has been met with criticism because it medically perpetuates the heteronormative orientation of society by failing to recog nise sex/gender diversity. Health insurance coverage for sex reassignment is thus bound to the diagnosis criterion ‘transsexual’, which means that transgender people must first clearly identify as either a woman or a man. This excludes people situated outside of the gender binary such as those who identify as queer or agender (Info box Annex) [51]. For this group, individual sex reassignment treatment measures targeting specific sex characteristics are either difficult or only possible if paid for privately [52].

This should change with the new guideline from 2018 titled ‘Gender Incongruence, Gender Dysphoria and Trans Health: S3 guideline for clinical diagnostics, counseling and treatment’ (Geschlechtsinkongruenz, Geschlechtsdysphorie und Trans-Gesundheit: S3-Leitlinie zur Diagnostik, Beratung und Behandlung), although so far, it is not binding for the MDS [53]. The S3 guideline allows much more scope for individualised treatment. Psychotherapy and the ‘Real-Life Test’ have been dropped as indication requirements for somatic treatments and informed consent has become the basis of treatment decisions. This should take the needs of transgender people better into account.

The discussion of variations of sex characteristics (intersex) is taking place between the two poles of medicalisation [54] and depathologisation. The WHO ICD-11, which will come into force in 2022, classifies intersex as a disorder, thereby implying a condition that fundamentally requires treatment. This classification has been criticised from a human rights perspective because it is based on a binary norm of sex, and variations from this norm are viewed as pathologic and as requiring treatment [55]. For this reason, the term ‘disorder of sex development’ (DSD) has been rejected and intersex described instead in terms of variations of sex characteristics [56].

Sex modifying surgery on intersex children that is not medically necessary can be traced to research from the 1950s on psychosexual development, which assumed that gender identity could be moulded by society. It was believed that an intersex child raised as a girl or boy would develop a corresponding identity and that this process could be assisted by surgery early in life [54, 57]. Surgery was often justified using the fear of stigmatisation and discrimination, fears based on a normative binary gender model in which intersex is a deviation. The birth of an intersex child was therefore considered a psychosocial emergency for parents. Social expectations and normative concepts of sex and gender held by medical staff and parents were also important [57].

In recent years, medical guidelines recommend restraint in regard to sex modifying surgery on intersex people. In a 2012 statement on the subject of intersex, the German Ethics Council stressed the need to carefully weigh up all the advantages and disadvantages of an intervention and its long-term consequences [58]. Similarly, a statement by the German Medical Association (Bundesärztekammer) entitled ‘The Care of Children, Youth and Adults with Variants/Disorders of Sex Development’ (Versorgung von Kindern, Jugendlichen und Erwachsenen mit Varianten/Störungen der Geschlechtsentwicklung) from 2015 makes it clear that surgery should only be performed if there is a serious threat to life or health [20]. Finally, the S2 guideline ‘Variants of Sex Development’ (Varianten der Geschlechtsentwicklung) from the German Society of Urology (DGU), the German Society of Pediatric Surgery (DGKCH) and the German Society of Pediatric Endocrinology and Diabetology (DGKED) from 2016 emphasises that sex modifying surgery on children incapable of giving consent be performed only in exceptional cases [59].

In 2019, the EU parliament passed a resolution calling for an end to medically unnecessary sex modifying surgery for intersex people [60]. In Germany, a draft bill protecting children from surgery changing sex characteristics has been in place since early 2020 [61]. This bill stipulates that sex modifying surgeries on children is only admissible if such surgery cannot be delayed or is necessary to avert a threat to life.

## 5. The health of LGBTI people

The selection of topics that follow is heavily dependent on the availability of findings from German and international studies. Although the estimates from international studies are not directly applicable to Germany, they do reveal important statistical associations and can give indications of possible health inequalities.

The groups included in the description below are intrinsically heterogeneous. Sex, gender and sexual orientation (Info box Annex) interact with other categories of social difference such as education, income, migration background/history or people of colour. The term ‘people of colour’ is a self-designation for people affected by racism [62]. Categories of difference are not understood as individual characteristics but as social power relations linked to privilege or disadvantage. They do not add up, yet in combination they produce specific life conditions which affect health. This interaction is described by the concept of intersectionality [63]. Thus, while the following focus is on gender and sex (transgender and intersex people) and sexual orientation (lesbian, gay and bisexual people), it must be noted that the heterogeneity of each respective group cannot be adequately portrayed with the available data. Health is not only dependent on sex, gender and sexual orientation, but on other categories as well.

### 5.1 Lesbian women

According to a survey on the health of lesbian women in Germany from 2005, 55% of respondents stated that they had good or very good subjective well-being. Only 1.1% reported poor physical health and 1.7% reported poor mental health [6]. A Swedish study found that the overall health of lesbian and bisexual women under 45 was worse than that of heterosexual women [64]. Particular aspects of lesbian health including physical illnesses (cancers), mental health (suicidality, mental illnesses), substance abuse and the experience of violence have been described [6, 65].

Conclusions on the cancer morbidity and mortality of lesbian women cannot be drawn from German cancer registry data. Findings from the few existing international epidemiological studies, in particular from the US, indicate either a higher incidence specifically for breast cancer among non-heterosexual women or no difference according to sexual orientation [66]. A Danish study has shown that the risk of developing cancer is not significantly higher for lesbian women in partnerships than for women in average. The incidence for cancers such as cervical, breast or lung cancer also roughly correspond to the expected incidence for women in general [67]. In contrast, data from the Danish National Cohort Study shows a higher cancer mortality rate for lesbian women than for heterosexual women (hazard ratio 1.62; 95% confidence interval (CI) 1.28–2.05) [68].

It is discussed that behavioural risk factors, particularly health behaviour (such as smoking, alcohol consumption [69–71] or reproductive behaviour [72]), the use of specific early detection measures [70], and the risk of sexually transmitted infections [73] play a role in the aetiology of these cancers. Until now, sexual contact between lesbian women has rarely been analysed in connection with sexually transmitted diseases. The results may also indicate a specific need for early detection.

In view of mental health, a systematic review of European studies showed evidence that the prevalence of risky substance use and/or addiction is higher in lesbian women compared to heterosexual women [69]. One of these studies found a 12-month prevalence of 14.0% (odds ratio 4.05; 95% CI 1.56–10.47) and a lifetime prevalence of 25.6% (odds ratio 3.43; 95% CI 1.60–7.33) for risky substance use and/or addiction in lesbian women compared to 2.9% and 7.1% for heterosexual women [74]. A further population-based, cross-sectional study from the USA analysed the 12-month prevalence of drug use and addiction. Lesbian women had significantly higher prevalences of marihuana use (16.7% vs 2.6%) and other drug use (12.6% vs 3.1%), and were more likely suffering from addiction to alcohol (13.3% vs 2.5%), marihuana (2.8% vs 0.2%) and other drugs (5.7% vs 0.4%), compared to heterosexual women [75].

In addition to health-related risk factors, there is also evidence that lesbian women engage in behaviour beneficial to health. In a German study from 2005, 77.6% of respondents stated that they regularly did physical exercise, and 40.7% said they were physically active two or more hours per week [6].

A number of studies show that lesbian women are at high risk of suicide, a fact which has received little attention in suicide prevention to date [68, 76]. Key influential factors in this context are experiences of violence and discrimination, particularly sexualized violence and violence at a young age [76, 77]. According to a Swedish survey, one of the few population-based studies that differentiated by sexual orientation, bisexual and lesbian women are the group most affected by discrimination, violence and threats of violence [64]. However, belonging to a sex/gender or sexual minority can also have a beneficial effect on health. In the US MetLife survey from 2006, 38% of respondents stated that supportive social networks had increased their resilience and ability to cope in a crisis, which in turn meant they were better able to deal with negative experiences [8].

### 5.2 Gay men

The health of gay men has so far been studied with a primary focus on sexually transmitted infections. This is due to the higher prevalence of HIV (human immunodeficiency virus) infection among gay men in comparison with heterosexual people or lesbian women. However, this focus risks reducing the life of gay men to their sexual behaviour. Comparatively little is known about the overall health of gay men. Data are available mainly for men who have sex with men (MSM), a group which includes gay and bisexual men as well as men who have sex with men but do not identify as gay or bisexual.

MSM face a higher risk of sexually transmitted infections compared to the general population. In the 2017 European MSM Internet Survey (EMIS), 14.2% of participants reported having ever been diagnosed with syphilis [78]. This risk was unevenly distributed within the MSM group: MSM diagnosed with an HIV infection reported a syphilis diagnosis more often (15%) than MSM without a diagnosis of HIV (3%). In 2018, a total 7,332 cases of syphilis were reported in Germany [79]. In cases with a known risk of transmission, 85.0% of these were attributed to MSM. Furthermore, 19.2% of EMIS participants reported having ever been diagnosed with gonorrhoea and 13.9% were ever diagnosed with chlamydia [78]. Here, too, men with an HIV infection more often reported a diagnosis compared to men without an HIV diagnosis.

Men who have sex with men also have a higher risk of HIV infection. In the EMIS study, 10% of all participants reported having been diagnosed with HIV. In Germany, the number of new HIV diagnoses among MSM has been decreasing since 2014 [80]. However, of the estimated 2,400 new HIV infections in 2018, 1,600 were attributable to MSM. MSM who are infected with both human papillomavirus (HPV) and HIV have a greater risk of developing anal and colorectal cancer because their immune system is suppressed [81, 82]. There is hope that measures such as improved tests, early treatment of HIV patients and HIV pre-exposure prophylaxis (PrEP) can further reduce the number of new HIV infections and consequently the number of reported comorbidities in the years ahead.

Homophobia and the criminal prosecution (in West Germany until 1994) of gay men can have an impact on mental health, health behaviour and physical health. Several international systematic reviews and meta-analyses show that gay and bisexual men, or MSM, are more likely to suffer from anxiety and depressive disorders, to be addicted to alcohol or drugs, or to be suicidal than heterosexual men [33, 83, 84]. According to an international meta-analysis from 2017, for example, the lifetime prevalence of suicidal thoughts for MSM is 35.0% (ranging from 13.2% to 55.8% depending on the country) [85]. Mental stress and suicidality can also be associated with an HIV infection. Available studies indicate that a positive HIV status leads to a significantly higher risk of suicidal thoughts [85]. Suicide attempts are also more common among gay and bisexual men compared to heterosexual people (relative risk 4.28; 95% CI 2.32–7.88) [84].

Mental illness and stress can also negatively impact healthy behaviour. A population-based cross-sectional study from the USA examined the 12-month prevalence of drug use and addiction. This showed that gay men have a slightly higher prevalence of heavy alcohol consumption (18.1% vs 13.7%), although this is not statistically significant [75, 86]. However, differences were more noticeable in regard to alcohol addiction (16.8% vs 6.1%), illegal drug use and drug addiction [75]. Possible reasons for this include the role of substances in coping with the stress of exclusion and discrimination experienced in connection with sexual orientation [33, 83]. The increasing social and legal recognition of gay people and ways of life is a positive development, that migt strengthen the health of gay men not least in the areas of mental health and health-related risk behaviour.

### 5.3 Bisexual people

The life and health situation of bisexual people is rarely considered as they are usually included in the groups of lesbian or gay people. The few available findings on bisexual people refer almost exclusively to women or men. Other sexes or genders such as non-binary or queer are very rarely taken into account.

There is less evidence of sexually transmitted infections in bisexual women compared to bisexual men. However, the frequency of such infections is rarely discussed in this group. In women who have sex with women, there is evidence of more frequent bacterial vaginosis [87]. With regard to bisexual men, an US survey showed that bisexual men are more likely to be HIV positive (7.7%) than heterosexual men (0.3%), but less likely to be so than gay men (17.4%) [88]. Another study found no differences between gay-identified or bisexual-identified men regarding other sexually transmitted infections [89].

A population-based cross-sectional study from the USA examined the 12-month prevalence of drug use and addiction among men and women. Levels of heavy alcohol consumption were higher among bisexual women compared to heterosexual women (25.0% vs 8.4%), and prevalence of marihuana use (22.2% vs 2.6%), other drug use (14.1% vs 3.1%) and alcohol addiction (15.6% vs 2.5%) was also higher [75]. Furthermore, bisexual people, particularly women, tend to consume significantly more tobacco than heterosexual, lesbian and gay people [90]. For bisexual men, there is a slightly higher prevalence of heavy alcohol consumption (16.4% vs 13.7%) than for heterosexual men. However, bisexual men have a higher prevalence of alcohol addiction (19.5% vs 6.1%), marihuana use (13.2% vs 6.2%), other drug use (17.7% vs 4.5%) or drug addiction (5.1% vs 0.5%) [75].

Bisexual people are not always accepted by society or by lesbian, gay and queer communities (Info box Annex), and are either not taken seriously in regard to their sexual orientation or face prejudice [91]. Like lesbian and gay people, bisexual people are at greater risk of suicidal thoughts and suicide attempts than heterosexual people [92]. Furthermore, a review has shown that bisexual people but also more generally, people who question their sexual orientation (‘questioning’), have a greater tendency to self-harm than lesbian or gay people [93]. Greater visibility and recognition within society could be beneficial for the mental health of bisexual people.

### 5.4 Transgender people

Transgender activism over the past few years has led to greater recognition of transgender people by the medical professions (Chapter 4.3). Despite this, the health situation for transgender people is still shaped by the heteronormative orientation of society and the medical professions [94]. Discrimination in everyday life as well as barriers to sex reassignment and healthcare in general (Chapter 4.3) are described as a burden by many transgender people and can lead to stress and poorer mental health.

International surveys indicate a high prevalence of depressive disorders among transgender people [4, 95]. Findings from New Zealand show that the 12-month prevalence of suicide attempts for young transgender people is nearly five times higher than for people who live in the gender assigned to them at birth (female/male) [96]. A review of international research literature from 2016 found high rates of non-suicidal self-harming behaviour in transgender people (17%–42%). The risk was particularly high for transgender people who identify as non-binary, i.e. as neither woman nor man (e.g. questioning, non-binary, agender; Info box Annex) [93]. These figures indicate an urgent need to raise social awareness of transgender people in order to reduce discrimination and thus promote transgender self-acceptance and mental health.

At present, little data is available on the sexual health of transgender people in Germany. International studies show that HIV prevalence is higher among transgender people than the overall population. A survey of transgender people in the USA revealed that 1.4% of the participants were HIV positive, compared to 0.3% of the overall population [97]. In addition, 46% of participants were unaware of their HIV status. The estimated HIV prevalence for transgender women in the USA was 14.1% (95% CI 8.7–22.2) and 3.2% for transgender men (95% CI 1.4–7.1) [98]. Another systematic review estimates global HIV prevalence for transgender women at 19.1% (95% CI 17.4–20.7) [99]. However, it is unclear to what extent the results of these studies can be transferred to Germany. Furthermore, the data on HIV prevalence for transgender people varies greatly between studies. Nevertheless, the figures clearly show that HIV can be an important health issue for transgender people.

With regard to physical activity, health behaviour of transgender people is also negatively influenced by institutional barriers. Exercise opportunities are usually geared towards a binary sex/gender norm, making access to public and private facilities such as swimming pools and sports clubs more difficult for transgender people. Furthermore, the anticipation and past experience of discrimination in organised sport is a barrier to beneficial health behaviour [100]. Some city-based civil society organisations offer exercise programs for transgender people and other sex/gender minorities. These can have a positive effect not only on physical health but also on mental health, as they provide an opportunity to interact and network with others [36].

### 5.5 Intersex people

Currently, there is scarce data on the overall health of intersex people. After many years of activism of intersex organisations, the discussion about the social and health situation of intersex people has received more public attention, particularly over the last decade. Sex modifying surgery on intersex people that is not medically necessary has been labelled a central health issue. Intersex organisations particularly criticise surgical and drug interventions that are performed without full, free and informed consent (for example on children) or aim to align intersex bodies with binary sex/gender norms, for example with the surgical alteration of intersex genitalia [55]. Medically unnecessary and procedures performed on children who are incapable of providing informed consent violate the right to physical integrity and self-determination [101].

Such measures are either irreversible or very difficult to reverse and can have serious health consequences such as infertility, chronic pain, incontinence, sexual dysfunction and mental disorders [23]. In a study of 78 adults from Hamburg, a large proportion of the respondents stated that medical treatment had been a negative experience. 62% of study participants showed signs of clinically relevant psychological stress, 47% reported having had suicidal thoughts and 13.5% reported episodes of self-harm in the past. The degree of psychological stress varied depending on the diagnosis [102, 103].

In recent years, guidelines have cautioned against any sex modifying surgery on intersex people that is medically unnecessary (Chapter 4.3). However, these guidelines are not binding, which may explain why the number of surgeries has not decreased. Secondary analysis of fixed-rate hospital treatment statistics (Diagnosis-Related Group statistics) from the Federal Statistical Office shows that the number of feminising and masculinising genital operations on children under the age of ten remained relatively constant between 2005 and 2016 [104, 105]. Refraining from medically unnecessary sex modifying surgery on children who are unable to give informed consent can help protect the rights of children as well as human rights [106, 107], while saving parents and healthcare personnel from making a premature decision in favour of potentially harmful procedures [105, 108].

### 5.6 Barriers to healthcare services

The Charter of Fundamental Rights of the European Union includes the principle of non-discrimination and calls for equal access to healthcare [109]. As described above, the issues relevant to lesbian, gay, bisexual, transgender and intersex people and the specific healthcare needs can vary considerably. Nevertheless, some general barriers to accessing adequate healthcare can be identified [110]: at the individual level, less use is made of screening and early detection services and in general, of medical and psychological services, or there is a delay in accessing these services [8]. Anticipated or previously experienced discrimination is a reason why people delay or do not to seek medical attention [111]. A Danish study, for example, showed that lesbian women are diagnosed with cervical cancer at a much more advanced stage than heterosexual women [67]. A lack of specialist knowledge and competencies among healthcare staff are also named as reasons for delaying medical attention [7, 65, 111, 112]. This can have negative consequences for health. Delayed use of healthcare services and misdiagnoses can lead to insufficient and inadequate care and poorer disease progression [67].

A further barrier to good healthcare is found at the level of interaction between service providers and patients [113]. A 2017 report based on a survey from the Federal Anti-Discrimination Agency describes discrimination against transgender and intersex people when physicians and healthcare staff refuse to recognise their sexuality, sex characteristics or gender identity [111]. The report also describes how health problems are not recognised because service providers partially attribute them to the patient’s sexual orientation, sex characteristics or gender identity. Prejudices and a lack of specialist knowledge of LGBTI-specific health issues can affect the use of healthcare services and the quality of care.

Structural, organisational and regional barriers to access can be seen at the level of the healthcare system. One problem is the orientation of care towards a sex/gender binary and a heterosexual norm [114]. Transgender women and men, for example, report that they have been refused gynaecological respectively urological examinations that are linked to their biological sex characteristics [111]. More areas in need of change are currently being identified with regard to the care of intersex children and adolescents. These areas include intersectoral co-operation within care provision, psycho-social support, and the design of the transition from paediatric and adolescent care to adult healthcare [115]. The National LGBT Survey from the United Kingdom indicates that the care needs of intersex people, particularly in the areas of psychotherapeutic and psychiatric care, have not yet been adequately addressed [116]. In general, there are also regional differences in the care provided. LGBTI advisory and care services that are based on sufficient specialist knowledge of sex, gender and sexual diversity are usually found in large cities and only extremely rarely in rural areas. Accessibility is therefore an issue, particularly for elderly people and those with limited mobility. This also applies to peer support centres and initiatives, which represent an important information resource for LGBTI people. The barriers described are significant because the care available has an impact on the health of LGBTI people and thus helps explain health inequalities.

## 6. Discussion

In recent years, civil society activists and organisations have achieved a greater degree of legal equality for lesbian, gay and bisexual people, a gradual depathologisation of transgender and intersex people, and a revision of medical guidelines for transgender and intersexual people. Nevertheless, sex, gender and sexual diversity have yet to gain full social acceptance and legal recognition. There are still strongly heteronormative social structures and cultural patterns that can affect the lives of LGBTI people and, as a consequence, their health. Services aimed at prevention, care and health promotion tend to be geared towards a sex/gender binary and a heterosexual norm. This affects the ability of LGBTI people to participate in society, the resources available to them and the levels of discrimination they face. However, with increasing social acceptance and legal equality, further improvements in terms of health equality for LGBTI people can be expected.

The available data on LGBTI health indicates that there are specific needs to be met. With regard to mental health, internalised negative attitudes towards one’s own sex characteristics, gender identity or sexual orientation can impede the development of a positive self-image. Gender and/or sexual orientation themselves are not the cause of higher prevalence of depression and suicidality. Rather, a lack of acceptance and experiences of discrimination and violence are psychologically stressful and can lead to mental illness. The health of LGBTI people would benefit from a social environment in which they feel accepted and supported together with an availability of peer support centres providing information, advice and networking opportunities. Overall, there is a lack of research on the factors that promote LGBTI health.

Moreover, there are many indications that healthcare may still be insufficiently adapted to the needs of LGBTI people and that expert knowledge and sensitivity when dealing with LGBTI-specific health issues are often lacking. This is particularly evident in the stable figures for sex modifying surgery on intersex children too young to provide informed consent, despite the fact that medical guidelines caution against interventions that are medically unnecessary. For this reason, greater consideration and recognition should be given to sex, gender and sexual diversity within healthcare.

In regard to the health issues described above, it should be noted that the majority of these are statistical estimates and probabilities which do not allow conclusions to be made about individuals. Furthermore, gaps in the data necessitated the use of international studies in addition to the findings from Germany. These international studies cannot be readily transferred to Germany due to different social constellations and other healthcare and social security systems.

Existing research on the individual groups is strongly focused on specific topics, with little information on LGBTI health in general. While research on sexual health primarily focuses on MSM due to the higher prevalence of HIV, there are large gaps in the data on the sexual health of other groups. There is also a need for in-depth research on physical and mental health to identify further health needs. Furthermore, many studies have their main focus on risk factors. In addition, a more detailed description and analysis of LGBTI health resources should be undertaken in order to better describe the everyday reality of LGBTI people.

Finally, in writing the present report we found it difficult to adequately portray the heterogeneity of each group within the LGBTI collective. The life situations of LGBTI people are influenced not only by sexual orientation and sex/gender, but also by other social categories (such as socioeconomic status [14], age [7], migration history and/or identification as a person of colour). More data is needed to account for the diversity within each group and to provide a basis for comparisons with the overall population. To increase the visibility of health and social inequalities and to underpin the discussion of areas where action is needed, specific attention must be given to LGBTI people in population-representative studies and monitoring systems.

A number of measures are currently being undertaken to gradually improve the information basis on LGBTI health in Germany. The change of Civil Status Act will increasingly go along with an altered recording of sex/gender in official health and social statistics. In the long term, larger population-based surveys should be designed to give a more differentiated account of sex/gender diversity. In the health monitoring studies at the Robert Koch Institute, such concepts are currently being tested, as are promising approaches to consider sexual orientations. Further examples include the Socio-Economic Panel of the German Institute for Economic Research (Deutsches Institut für Wirtschaftsforschung) that included questions on sexual orientation for the first time in 2016 [1], and the study Health and Sexuality in Germany (Gesundheit und Sexualität in Deutschland) of the Federal Centre for Health Education [13].

Information is also needed on specific concerns and on the living conditions of LGBTI people. The Queergesund*-Study, for example, carried out a participatory needs analysis on the promotion of health for lesbian, bisexual and queer women. It identified an extensive need for action relating to social anti-discrimination policy, reducing heteronormativity in healthcare and support for LGBTI people and women’s/lesbian community projects as a central health resource. Currently, the Federal Ministry of Health funded InTraHealth project (2019–2022) is dedicated to improving access to healthcare for intersex and transgender people by reducing discrimination on the part of care providers that can be a barrier to access [117]. This project will expand the existing LGBTI information portal (Wissensportal LSBTI^2^) to include an interactive self-learning module on intersex and transgender that is aimed at healthcare professionals. Furthermore, the European Union Agency for Fundamental Rights (FRA) conducted a second Europe-wide survey on LGBTI people’s experiences of discrimination in 2019, the results will be published in 2020 [118]. This kind of information is urgently needed to identify the healthcare needs of different groups and to promote health equity.

It is essential that the findings of studies are always viewed in their social context and that besides health risks also resources that promote health need to be considered. This can help prevent discrimination and stigmatisation of LGBTI people through the way of health reporting. Moreover, health reporting that is sensitive to discrimination should involve the concerned subjects in the design of the study, the survey itself, and the evaluation and presentation of results.

## Key statements

- Sex, gender, sexual orientations and ways of living are diverse.
- The heteronormative orientation of society represents a health risk for LGBTI people.
- While progress has been made on legal equality for homosexual and bisexual people both in Germany and internationally, this is still underdeveloped for transgender and intersex people.
- LGBTI people face specific barriers to healthcare access.
- The data basis on the health of LGBTI people should be improved.

## Figures and Tables

**Table 1:** Sexual orientations in Germany. Results of population-wide interview surveys Source: Own table

	Proportion	Source
**Cohabitating same-sex partnerships**		
Around 94,000 people	0.5%–0.9% of all couples sharing a household	Destatis Microcensus 2015 [14]
**Self-identification as lesbian, gay or bisexual**		
Total	1.9%	DIW SOEP 2016 [1]
<45 Years	2.8%	
>60 Years	Under 1%	
Women, 21–25 years	Around 3% lesbian Around 6% bisexual	BZgA Jugendsexualität (Youth sexuality) 2015 [11]
Men, 21–25 years	Around 5% gay Around 2% bisexual	
Women, 18–75 years	1.3% lesbian 1.8% bisexual	BZgA Liebesleben (Love life) 2017 [13]
Men, 18–75 years	2.1% gay 1.4% bisexual	
**Close, same-sex body contact in the last twelve months**		
Women, 21–25 years	Around 14%	BZgA Jugendsexualität (Youth sexuality) 2015 [11]
Men, 21–25 years	Around 12%	
**Self-description as not exclusively heterosexual**		
Women, ≥14 years	Around 11%	University of Leipzig Sexualverhalten in Deutschland (Sexual behaviour in Germany) 2016 [15]
Men, ≥14 years	Around 10%	
Women, 18–75 years	22.4%	BZgA Liebesleben (Love life) 2017 [13]
Men, 18–75 years	13.8%	

BZgA = Federal Centre for Health Education, Destatis = Federal Statistical Office, DIW = German Institute for Economic Research, SOEP = German Socio-Economic Panel

## Annex Info box: Explanation of terms

### LGBTI

The LGBTI acronym encompasses a wide range of sexual orientations, ways of living, sexes and genders: **l**esbian, **g**ay, **b**isexual, **t**ransgender and **i**ntersex.

Queer is another collective term that can include gender identities and sexual orientations outside of the heterosexual gender binary. Queer is not considered in this article due to the great heterogeneity within the group and the insufficient data available.

### Coming out

In a society in which a norm of two sexes and heterosexuality dominates, many lesbian, gay, bisexual and transgender people go through a long phase of developing an internal awareness of their gender identity and/or sexual orientation. Informing other people of their gender identity/sexual orientation is called coming out. Coming out remains a life-long process, as there are always situations where the decision must be made whether or not to reveal the persons gender identity/sexual orientation to others. For intersex people, coming out involves telling other people that their body does not fit the medically established norms of female and male.

### Sexual orientation and ways of living

Sexual orientation describes which genders a person is attracted to, with whom they have sexual contact and whether this also forms part of their identity. Sexual desire does not necessarily translate into corresponding sexual behaviour or sexual identity. A woman who has sex with women, for example, may not necessarily consider herself lesbian or bisexual.

Homosexual people feel romantically and sexually attracted to people of the same sex/gender. Some people reject the term homosexual because it focuses too much on sexuality and not enough on emotional aspects and ways of living. For this reason, the terms lesbian and gay are more common. Lesbian describes women who are romantically and sexually attracted to women, while gay men feel romantically and sexually attracted to men.

Bisexual people have romantic and sexual relationships with both women and men. Some also use the term bisexual to express that they are attracted to people of different sexes/genders. This can include transgender and intersex people as well as women and men. The term pansexual is also used to stress an attraction to people of different sexes/genders.

People are heterosexual if they identify as a woman or a man and feel attracted to people of the opposite sex.

### Sex/gender

The concepts of gender and sex encompass two dimensions: one that is socially constructed and one that is based on biomedical characteristics [119, 120]. Both dimensions exert influence on one another [121]. The biological dimension of sex comprises genetic, anatomic, physiological and hormonal characteristics. The social dimension of gender relates to social concepts of gender that influence cultural conventions, social roles and identities. On an individual level, people can perceive they have one or no gender in comparison with social norms. There is a great variety within both dimensions of sex and gender [122–124], which means that both must be understood as non-binary.

Intersex people are born with genetic, anatomical or hormonal variations in sex characteristics [125]. These variations may be visible at birth, become apparent later in life, or remain undetected throughout life. Intersex is not a third sex, but a diverse set of congenital variations in sex characteristics. The label ‘Disorders of Sex Development‘ (DSD) has been rejected by many intersex self-advocacy groups because it describes these congenital variations in terms of a medical disorder. Intersex people can have different gender identities and sexual orientations.

People do not necessarily identify with the biological sex they are born with. Gender identity describes whether a person feels they are a woman, a man, a sex in-between the two, whether they identify as being outside these two categories (e.g. questioning, non-binary), or whether they identify as having no gender (e.g. agender). There are very different self-designations for different gender identities. This article also focuses on transgender, an umbrella term for diverse gender designations (and self-designations) by people who do not or not entirely identify with the sex they were born with (trans identity, transgender, trans woman, trans man, etc.).
